# Regulatory Requirements for the Quality of Allergen Products for Allergen Immunotherapy of Food Allergy

**DOI:** 10.1007/s11882-021-01008-9

**Published:** 2021-05-10

**Authors:** Lisa Englert, Vera Mahler, Andreas Bonertz

**Affiliations:** Paul-Ehrlich-Institut, Langen, Germany

**Keywords:** Allergen immunotherapy, Food allergy, Quality requirements, Allergen regulation

## Abstract

**Purpose of Review:**

Medicinal products for allergen immunotherapy (AIT) of food allergies have gained enormous momentum in recent years. With this new class of products entering marketing authorization procedures, compliance to regulatory requirements becomes a critical element. Here, an overview is provided on specific requirements and aspects concerning the quality control and manufacturing of these products.

**Recent Findings:**

Recent developments in the field of AIT for food allergies are divers, including products for oral, epicutaneous, and subcutaneous application, most notably targeting egg, milk, and peanut allergy. As the source materials for food AIT product are typically produced for food consumption and not for medicinal purposes, unique challenges arise in the manufacturing processes and controls of these medicinal products. Individual approaches are needed to assure acceptable quality, including control of relevant quantitative and qualitative characteristics. Major characteristics for quality verification include determination of protein content, total allergenic activity, and major allergen content. The applied manufacturing processes need to be established such that relevant process parameters are kept within justified limits and consistency of produced batches is assured.

**Summary:**

Allergen products for food AIT present specific challenges with respect to quality aspects that differentiate them from other commonly available AIT products. While established regulation is available and provides clear guidance for most aspects, other issues require consideration of new and individual settings relevant here. Consequently, as experience grows, respective amendments to currently available guidance may be needed.

## Introduction

According to European laws, allergen products are medicinal products, and as such, they typically require a marketing authorization to be allowed in the market. Individual allergen products targeting various allergies are available in the European Union (EU). While the availability of authorized allergen products is dependent on the specific member states [1], most authorized AIT products are directed at respiratory allergies, for example, pollen from the birch group, sweet grasses, or house dust mites. In addition, AIT products for the treatment of insect venoms are widely available. This situation differs severely when looking at the availability of AIT products for the treatment of food allergies. While first authorizations have been granted in the US [2], no AIT product for the treatment of food allergy had been authorized in Germany, until very recently the first product for oral AIT of peanut allergy has been approved in a centralized procedure. This evident lack of approved products is observed despite Germany being one of the largest markets for AIT in the EU. While for diagnosis of food allergy a broad panel of authorized products is available in many cases, treatment of food allergies is traditionally performed in clinical practice by individual and non-industrial preparation of an AIT product directly from the food source [3, 4]. This preparation is then applied to the respective patient via oral immunotherapy. While this approach allows a comparably quick route to treat an individual patient with a patient-tailored product, it does inherit some crucial drawbacks. For example, such products are typically much less controlled and standardized as would be the case for AIT products that are produced under the provisions of good manufacturing practice (GMP) and for which quality, safety, and efficacy have been independently assessed by a regulatory body [3, 5].

In recent years, developments in the field of AIT for food allergies, most notably targeting egg, milk, and peanut allergy, have gained enormous momentum. Several large clinical studies have been published looking at AIT products for peanut allergy [6•, 7] . Currently published approaches include products with minimal modifications from the typical food product (e.g., peanut flour) for oral (OIT, e.g., [8]) or sublingual immunotherapy (SLIT) [9, 10], products that are processed to be applied for epicutaneous immunotherapy [11•, 12], as well as products that have undergone chemical modification for subcutaneous immunotherapy (SCIT) [13••, 14]. Some of these developments are already advanced to a level where they are being assessed for marketing approval.

While general overviews on the development of food allergen immunotherapy have been published elsewhere (e.g., [15••, 16, 17]), in this review, we will discuss noteworthy regulatory and quality aspects in the field of AIT for food allergy that have become relevant in recent years and where much development has occurred. Development, regulation, and assessment of AIT products for food allergies is a highly dynamic field at the moment, especially considering differing approaches with regard to all of these aspects followed in different regions of the world. Overall, the approach followed by the FDA concerning quality regulation of food allergen products is similar to the requirements applicable in the EU. Yet, some differences remain, for example, concerning specific requests on necessary analytical procedures as well as the FDA’s approach to standardization in contrast to the use of IHRPs in the EU. For a comprehensive overview on requirements requested by the FDA, partially also in comparison to the EU, we refer to the review by David et al. [18]. For the sake of clarity, we will here only consider relevant aspects in the EU.

## Quality Requirements for AIT Products in Food Allergy

To achieve a marketing authorization for any medicinal product, the manufacturer of that product must provide detailed information on various quality and manufacturing aspects. For this, it needs to be demonstrated that the product can be produced in a consistent and reliable quality. Several regulations and guidelines need to be considered and followed to confirm these requirements.

The European Pharmacopoeia (Ph. Eur.) defines one such crucial set of rules. The Ph. Eur. is a collection of pharmaceutical rules developed by the European Directorate for the Quality of Medicines & Healthcare (EDQM). These rules are mandatory to be followed by manufacturers. The Ph. Eur. includes monographs on specific medicinal products (such as the monograph on allergen products [19]) as well as on pharmaceutical test methods. The monograph on allergen products applies to all allergen products manufactured from natural sources and describes specific requirements on various manufacturing aspects and acceptance criteria for the control of such products (Table 1). This includes requirements on the source materials, an In-House Reference Preparation (IHRP) (which is a representative preparation of the active substance used to verify batch-to-batch consistency), as well as specific requests on tests to be performed on the product. This includes verification of the identity of the product as well as requests on the confirmation of consistent quantitative parameters of the medicinal product. Such parameters include the determination of the protein content, total allergenic activity, and the content of relevant allergens. For specific tests, the monograph states maximum limits that may be applied as acceptance criteria (e.g., protein content may not exceed a range of 50 to 150% of a stated content). While some of these acceptance ranges appear comparably wide, they were introduced to account for the biological variability of the source material that is introduced into the production process. It should be noted however that the acceptance criteria and specifications set for an individual test must be justified by the manufacturer. In consequence, for a product where such wide acceptance criteria are not justified as the source material already shows high consistency with regard to specific quality aspects (e.g., protein content), more restricted acceptance criteria need to be applied.

**Table 1:** Selection of critical quality attributes tested for food AIT products

Attribute	Test	Method (example(s))	Typical acceptance criteria
Identity	Protein profile Allergen profile	SDS-PAGE, HPLC Immunoblot	Corresponds to IHRP, relevant allergens are present^1^
Content	Protein content	Kjeldahl, Bradford	max. 50–150% of the stated amount^1^
	Total allergenic activity	Competitive IgE-binding test using sera from allergic patients	max. 50–150% of the stated amount^1^
	Individual allergens	Binding tests using allergen-specific antibodies, mass spectrometry	max. 50–200% of the stated amount^1^
	Aflatoxins	LC	B1: ≤ 2.0 ppb Sum of B1, B2, G1, and G2: ≤ 4.0 ppb^2^
	Microbiological contamination/sterility	Membrane filtration, Pour-plate method	*Non-aqueous preparations for oral use:* total aerobic microbial count (TAMC): < 1000 CFU/g Total yeast and molds count (TYMC): < 100 CFU/g^1^
	Particle size distribution	Analytical sieving, laser diffraction	Product-dependent particle sizes at the undersize values of 10 percent, 50 percent, and 90 percent (x_10_, x_50_, and x_90)_

^1^According to Ph. Eur. , ^2^According to Commission Regulation (EC) No 1881/2006

Supplementing regulations of the Ph. Eur., additional guidelines are relevant and need to be considered for the assessment of AIT products for food allergy. These include guidelines from the International Committee for Harmonisation (ICH) that address various quality aspects, including, but not limited to, issues on the validation of the manufacturing process, the validation of analytical methods to demonstrate their suitability for their intended purpose, as well as guidance on stability assessment to reliably determine the shelf life of any given medicinal product. For allergen products, especially the EMA Guideline on Allergen Products: Production and Quality Issues [20] introduced precise requirements to allow in-depth characterization of allergen products.

## Regulatory Challenges for OIT Quality Assessment

It has become evident, however, that available regulations and guidelines only marginally considered allergen products for food AIT. As the currently available and authorized AIT products primarily aim at treating aeroallergens and insect venom allergies, available guidance considers these products far more specifically than food AIT products.

This is illustrated, for example, when looking at a typical production process for a food AIT product. In cases where the food is administered via oral immunotherapy, there may only be very limited steps in the manufacturing process from the source material to the final product. As a result, the active substance effectively is very similar or even identical to the source material. However, according to GMP guidance in force, the production of the active substance needs to be performed within the regulations of pharmaceutical GMP. Yet, the question becomes relevant, at which step a food source material must be manufactured and controlled according to pharmaceutical GMP regulation. According to EU-GMP guidelines [21], the production process for biological medicinal products should be validated and follow pharmaceutical GMP. Such production steps include steps for pre-treatment or manipulation of particle size (e.g., milling), with the stringency of GMP in active substance manufacturing increasing as the process proceeds from early steps to final steps, purification, and packaging. Yet, typically, source materials for food AIT derive from suppliers that provide their products for food consumption. For example, in the case of peanuts, this could typically be the shelled peanuts. However, additional manufacturing steps may follow, like roasting, grinding, defatting, or sieving steps. As these manufacturing steps may have a considerable impact on the product’s quality characteristics, they need to be controlled adequately [22]. For instance, the impact of roasting on allergenic properties of peanut proteins is well-known [23]. However, during manufacture of a peanut-derived product for consumption as food, corresponding process parameters are predominantly established focused on the aim to achieve a food with desirable purity, color, and taste. For use of such a pre-processed food as an active substance for food AIT, it is crucial that relevant process parameter ranges of the food process are explored on their possible effect on product consistency especially with respect to allergenic properties. This is essential as the intended objective of use is fundamentally different for a medicinal product and consequentially requires adapted parameters to consider. Based on the outcome of these studies, it has to be assured that relevant process parameters are kept within justified limits if the food material is intended to be rededicated for use in the manufacture of a medicinal product. This is considered especially important for certain types of foods where not all components of the active substance may be analytically accessible for release testing. For example, for release testing of a nut flour, an aqueous extract of the flour will have to be made to determine the potency of the protein profile of a respective batch. However, in this case, hydrophobic allergens may not be extracted, and their presence and content may thus be not correctly monitored. Besides, consistent quality of an allergen product is largely determined by the selection of adequate starting material. While for development for more typical medicinal products, dedicated and qualified starting materials for use in pharmaceutical processes are generally chosen, and the situation may be more challenging for a food intermediate which was primarily not intended for use as active ingredient in a medicinal product. If, for example, different species, cultivars, or mixtures thereof are included, it has to be assured retrospectively that they are sufficiently comparable in their allergenic attributes and representative for the allergenic agent, i.e., commonly consumed in the countries where the product is intended to be marketed.

Further challenges arise for the analytical characterization of food allergens. Commonly, available AIT products containing aeroallergens are produced involving an extraction process, resulting in an aqueous solution containing protein and non-protein components. From these aqueous solutions, various testing can be performed, for example, determination of the total allergenic activity by IgE ELISA inhibition assays. However, in the manufacturing process for food allergen products, a comparable extraction process may not be applicable. Food allergens in OIT are commonly administered in the form of a flour that is mixed into a vehicle food to be then ingested by the patient. While patients are thus administered all allergenic components in a comparable composition as they are exposed to in their daily life, in consequence, the active substance, as it will be formulated into the final drug product, will not be directly accessible in a form that allows application of commonly available techniques for some relevant characterization methods such as determination of the biological potency (e.g., IgE reactivity) or the quantification of relevant allergens. Where the active substance must be prepared specifically for such testing (e.g., by solubilizing in an aqueous solution), this may result in a bias as the composition of the components in the solution may not be representative of the composition in the drug substance itself. For example, relevant lipophilic components (which, e.g., constitute major parts of nuts) that may be relevant for the allergenic potency of the product and that are present in the drug substance itself may be severely underrepresented in the aqueous solution to be analyzed. Manufacturers must account for such problems by combining various analytical methods and by providing documentation on a full validation for each individual method. This includes a justification for the choice of the extraction medium based on data obtained in comprehensive extraction studies with, e.g., different buffers, extraction times, and temperatures. Thereby, it has to be taken into account that while some extraction media may on the one hand efficiently extract most of the allergens, these media might on the other hand not be suitable for use in an immunoassay [24]. Thus, several different extraction set-ups may have to be established considering the extraction efficiency with respect to relevant allergens and with respect to the particulars of each individual analytical method applied. Nevertheless, such predicament may leave some gaps in the full understanding of the qualitative characteristics for these products.

Last but not least, allergen products for therapeutic use are generally defined by a biological potency which is also reflected in the labelled strength of the medicinal product. This labelled strength is typically presented in manufacturer-specific biological units that are not directly comparable to products from other manufacturers. However, some food AIT products are commonly standardized and labelled based on the protein content [25]. In this case, a correlation between the biological potency, mostly determined by a competitive IgE-binding test, and the protein content has to be demonstrated to assure that the labelled strength (in this case the protein content) is still indicative for the allergenic potency of the product [20].

It is of crucial importance that a product of controlled and consistent quality is supplied to the patient. For both, an initial dose escalation as well as for the following maintenance phase, it needs to be assured that the qualitative and quantitative parameters of an OIT product are in a well-controlled range. If, for example, severe fluctuations in the biological potency or the content of relevant allergens occurs from one batch to another, this may lead to a situation that a patient, without his or her knowledge, ingests a product that may have a strongly increased potency as compared to the previous dose. Such scenario would potentially be accompanied by a considerably higher risk of experiencing severe adverse events, which may become especially relevant in case of OIT with very potent food allergens like nuts [26, 27]. Taking into account that patients typically apply their OIT products at home (after an initial dose escalation under clinical supervision), such risk is even more critical. Narrowing of the allowed acceptance range for parameters such as the total allergenic activity may be necessary as a result of this.

## Conclusions

With developments happening fast, new knowledge, experience, and technical advances need to find their way into current regulatory guidelines and requirements. For some aspects, this is already happening. According to the current version of the Ph. Eur. Monograph on allergen products [19], there are specific requests on the characterization of the first In House Reference Preparation (IHRP), which is a batch of the active substance that is produced and used for the subsequent characterization of the medicinal product and to assure batch-to-batch-consistency for future batches to be produced. The monograph states that the biological potency of the first IHRP is determined in patients by in vivo techniques such as skin testing and expressed in units of biological activity. Exceptions are only allowed in cases where there are not enough patients available to perform such testing. While such procedure may result in the confirmation that the active substance contains allergenically active components (as being visualized by the corresponding skin reaction), it can be quite burdensome for the patient, and the information gained on the actual medicinal product is very limited. For example, as only few patients are tested [28, 29], it is highly questionable that such patient populations allow reproducible results on the actual potency. In addition, the route of administration is completely different (skin prick test as compared to epicutaneous, oral, or sublingual administration). The committee responsible for the monograph on allergen products has acknowledged this situation and proposed respective changes that allow manufactures to characterize their products on the basis of in vitro methods. While these changes are essential, it has become evident that current guidelines only marginally consider development and characterization of allergen products for AIT of food allergy, leaving room for uncertainties and misinterpretation. With the relevance of these products increasing, it may be inevitable that current guidelines are amended or that new guidelines are developed to more specifically consider these products in more detail. In any case, we are at the dawn of an era where high-quality products for AIT of food allergy in line with current state of the art for quality, safety, and efficacy finally become available to the patients in need.
